# 

**Published:** 1886-03

**Authors:** 


MARRIED.

In Galveston, January 19, 1886, Dr. H. P. Cooke, City Physician, to Mrs. Carrie Evans, daughter of the late Willard Richardson, Esq.

On the 11th of January, 1886, Dr. G. W. Radford, of Hookerville, Burleson county, Texas, to Miss Willie Williams, of Caldwell, a sister-in-law of Dr. H. H. Darr, Ex-First Vice President Texas State Medical Association.

Dr. Z. W. Baker, our genial friend and subscriber at Temple, took unto himself a fair bride on the 6th inst. We tender our congratulations.

